# Barriers to genetic testing in clinical psychiatry and ways to overcome them: from clinicians’ attitudes to sociocultural differences between patients across the globe

**DOI:** 10.1038/s41398-022-02203-6

**Published:** 2022-10-11

**Authors:** Justo Pinzón-Espinosa, Marte van der Horst, Janneke Zinkstok, Jehannine Austin, Cora Aalfs, Albert Batalla, Patrick Sullivan, Jacob Vorstman, Jurjen J. Luykx

**Affiliations:** 1 Sant Pau Mental Health Group, Institut d’Investigació Biomèdica Sant Pau (IBB-Sant Pau), Hospital de la Sant Creu i Sant Pau, Universitat Autònoma de Barcelona, Barcelona, Catalonia Spain; 2 Department of Medicine, School of Medicine, University of Barcelona, Barcelona, Spain; 3 Department of Clinical Psychiatry, School of Medicine, University of Panama, Panama City, Panama; 4 Department of Mental Health, Parc Tauli University Hospital, Institut d’Investigació i Innovació Parc Tauli (I3PT), Sabadell, Barcelona, Spain; 5 Department of Psychiatry, Brain Center Rudolf Magnus, University Medical Center Utrecht, Utrecht University, Utrecht, the Netherlands; 6 Department of Translational Neuroscience, Brain Center Rudolf Magnus, University Medical Center Utrecht, Utrecht University, Utrecht, the Netherlands; 7 Outpatient Second Opinion Clinic, GGNet Mental Health, Warnsveld, The Netherlands; 8 Department of Psychiatry, Radboud University Medical Center, Nijmegen, The Netherlands; 9 Karakter Child and Adolescent Psychiatry, Nijmegen, The Netherlands; 10 Department of Medical Genetics, University of British Columbia, Vancouver, BC Canada; 11 Department of Psychiatry and Medical Genetics, Genetic Counselling Training Program, University of British Columbia, Vancouver, BC Canada; 12 Department of Clinical Genetics, University Medical Center Utrecht, Utrecht, The Netherlands; 13 Center for Psychiatric Genomics, Department of Genetics and Psychiatric, School of Medicine, University of North Carolina at Chapel Hill, Chapel Hill, NC USA; 14 Karolinska Institute, Stockholm, Sweden; 15 The Centre for Applied Genomics, Program in Genetics and Genome Biology, The Hospital for Sick Children, Toronto, ON Canada; 16 Department of Psychiatry, Hospital for Sick Children, Toronto, ON Canada; 17 Department of Psychiatry, University of Toronto, Toronto, ON Canada

**Keywords:** Medical genetics, Predictive markers

## Abstract

Genetic testing has evolved rapidly over recent years and new developments have the potential to provide insights that could improve the ability to diagnose, treat, and prevent diseases. Information obtained through genetic testing has proven useful in other specialties, such as cardiology and oncology. Nonetheless, a range of barriers impedes techniques, such as whole-exome or whole-genome sequencing, pharmacogenomics, and polygenic risk scoring, from being implemented in psychiatric practice. These barriers may be procedural (e.g., limitations in extrapolating results to the individual level), economic (e.g., perceived relatively elevated costs precluding insurance coverage), or related to clinicians’ knowledge, attitudes, and practices (e.g., perceived unfavorable cost-effectiveness, insufficient understanding of probability statistics, and concerns regarding genetic counseling). Additionally, several ethical concerns may arise (e.g., increased stigma and discrimination through exclusion from health insurance). Here, we provide an overview of potential barriers for the implementation of genetic testing in psychiatry, as well as an in-depth discussion of strategies to address these challenges.

## Introduction

Genetic testing has evolved rapidly over recent years [1]. New technologies in genetic testing provide important new information about the diagnosis, treatment, and prevention of diseases and are of great value for precision medicine [2–4]. Nonetheless, at the time of writing, a range of barriers impedes such tests from being implemented in clinical psychiatry [5–7]. This review addresses the current state of genetic testing in psychiatry and lists recommendations on how to overcome such barriers. We first address general aspects of genetic testing, mainly its potential clinical yield. We then briefly discuss methods and applications of genetic testing in psychiatry, followed by a review on barriers to genetic testing as well as proposed ways to overcome them.

Indications for genetic testing vary by disorder. Given the current evidence and its widespread professional support we highlight examples of clinical testing indications for autism spectrum disorders (ASD). However, evidence to support direct-to-consumer testing will require further investigation for all psychiatric disorders. Regarding polygenic risk scoring (PRS) and pharmacogenetics, evidence is increasing rapidly, with high potential for future clinical translation of both, such as for diagnostic purposes and pharmacological interventions [8, 9].

## Potential of genetic testing in clinical settings

To date, genetic testing has been implemented most extensively in oncology and cardiology. For example, multigene panel testing for hereditary cancer predisposition, including breast, ovarian, and colorectal cancer, has been readily incorporated into clinical practice [10–12]. Due to the extensive overlap in cancer phenotypes and genetic heterogeneity, the use of panels containing a broad variety of hereditary cancer genes can have high clinical validity and improve risk assessment, early detection, and prevention of cancer [13, 14]. For already diagnosed patients, genetic panel testing can provide useful information for treatment decision-making [15]. Therefore, recommendations have been made to extend the use of genetic testing in oncology and include it as standard of care [15].

In cardiology, DNA-sequencing is widely used for the diagnosis and clinical management of heritable heart diseases, such as hypertrophic cardiomyopathy and long QT syndrome, with a diagnostic yield of genetic testing in the range of 30–50% and 60–70%, respectively [16]. Recent studies have also reported a potential role for PRS in cardiology. For example, in predicting coronary artery disease, it has outperformed any single traditional risk factor [17]. How psychiatry may benefit from the experience with clinical translation of PRS gained in other fields of medicine was recently reviewed elsewhere [18].

Oncology and cardiology are leading fields in the implementation of pharmacogenetic testing. The Clinical Pharmacogenetics Implementation Consortium (CPIC) has produced prescribing guidelines for various drugs according to *CYP2D6*, *DPYD*, and *TPMT* genotypes in oncology [19–21], and *CYP2C19*, *CYP2C9*, *SLCO1B1*, and *VKORC1* genotypes in cardiology [22–24].

In psychiatry, genetic testing can be used to diagnose underlying genetic syndromes (e.g., 22q11.2 deletion syndrome) and—in research settings—to provide insight into prognosis and treatment response, particularly for disorders with high heritability estimates, such as ASD, attention deficit and hyperactivity disorder, schizophrenia, and bipolar disorder [25, 26]. The underlying causes of these disorders are often elusive, resulting in a range of diagnostic and prognostic uncertainties for patients and families. Identifying a genetic condition underlying the diagnosis can help clarify medical risks associated with the diagnosis, test family members at risk for the condition, and avoid unnecessary testing, particularly in ASD [27–31]. Additionally, genetic testing may provide information to identify, classify, and discriminate between different stages of disease or patient subtypes, thereby contributing to the objective of personalized patient care [32–34]. In research settings, genetics has also been shown to help identify prognostic factors, although their clinical applicability has remained unresolved so far [35]. Furthermore, genetic variation in drug response (pharmacogenomics) has been widely investigated: while evidence supports lower chances of drug-gene interactions for patients undergoing pharmacogenetic testing, effects of such genetic testing on remission rates have remained unclear [36]. In line with such findings, the CPIC has issued guidelines on the dosing of antidepressants according to *CYP2C19* and *CYP2D6* genotypes [37, 38]. However, with the advance of technology and new methodologies, focus has shifted from targeted *CYP* genotyping to genome-wide association studies (GWASs) as an important source of pharmacogenetics data. GWASs have proven successful in identifying complex pharmacogenomic traits in medicine, including psychiatry [39]. The largest GWAS of antidepressant response to date found that SNP-based heritability is significantly different from zero, although currently the power to predict such a response in other cohorts using whole-genome data seems limited [40]. Finally, genetic testing may also be a valuable part of multi-omics approaches, including neuroimaging, digital phenotyping, and computational models, when aiming to perform multimodal analyses of predictions for diagnosis, prognosis, and treatment response in psychiatry [41–43].

### Should we move from targeted genetic testing to broad genetic testing?

Targeted genetic testing may be done to confirm a suspected diagnosis based on phenotypical or clinical features, family or personal medical history, such as in Duchenne muscular dystrophy and Fragile X syndrome [44, 45]. Using targeted genetic testing, a clinician aims to uncover whether an a priori hypothesized genetic etiology of a specified disease entity is present. In broad genetic testing, the disease entity is not pre-specified, but the clinician still suspects a genetic etiology of the clinical presentation. An example of broad genetic testing is whole-genome sequencing (WGS, sequencing of the entire genome) to examine a possible underlying genetic etiology in ASD (the current yield being around 10% in ASD) [46].

While targeted genetic testing answers a defined hypothesis (“*this* genetic etiology”), broad genetic testing addresses the question of genetic causation more broadly (“*a* genetic etiology”) [47]. Broad testing has an increased probability of revealing incidental findings—which is the subject of ongoing debate about the consequences for patients and their families, interpretation of results, usefulness for research, and ethical, financial, and political concerns [48].

As next-generation sequencing gradually becomes less expensive, WGS and whole-exome sequencing (WES; sequencing the ~1% coding part of the genome) are becoming more and more feasible options in clinical practice [49]. However, cost-effectiveness has not yet been fully established and is likely to vary according to the clinical setting; [49, 50] for example, genetic testing is likely to be more cost-effective in neonatology than in family medicine settings.

### Readiness—what is an appropriate test?

With ever-evolving technologies, it is essential to monitor and continuously evaluate whether tests meet the requirements to be considered sufficient to be implemented in clinical practice [51]. In general, genetic tests are assessed on the basis of four main topics: (1) analytical validity: the ability to accurately and reliably measure the genotype of interest—this is usually done by testing the sensitivity and specificity of the test; (2) clinical validity: the ability to accurately and reliably detect or predict a clinical condition—in addition to sensitivity and specificity, the positive and negative predictive values (PPVs and NPVs, respectively) of a test are examined; (3) clinical utility: the comparison of risks and benefits, and the assessment of clinical usefulness—this involves consideration of efficacy, effectiveness, and safety; and (4) ethical, legal, and social implications [48, 51–56].

ASD and intellectual disability (ID), collectively referred to as neurodevelopmental disorders (NDD), at present qualify as the only psychiatric disorders with enough evidence supporting genetic testing as part of standard clinical practice. Chromosomal microarray analysis (CMA) has been offered as a diagnostic tool for developmental delay as well as ASD for some years (for an example of a description with clinical indications, see cited references) [57, 58]. Nowadays, WES is recommended as first-tier clinical genetic diagnostic tool for NDD [59], with discussions ongoing for the incorporation of WGS as the first-choice genetic test in NDD [60]. Nonetheless, studies suggest low adoption rates of such tests in clinical practice [61]. For pharmacogenomics, important initiatives were recently launched in Europe with the funding of a large pharmacogenomics project for psychotropic medications by the EU Horizon 2020 program [62, 63].

Furthermore, when evaluating the clinical utility of genetic tests, special consideration must be given to risk. The effect size of risk (or resilience) on a group level, traditionally represented as the odds ratio (OR), must be translated to measures of individual risk, such as PPVs and NPVs. Although group- or population-level effect sizes may appear substantial, their clinical translation requires the application on an individual level, i.e., a translation that represents the individual risk of the patient, rather than the complete at-risk population [64].

## Methods and applications for genomic testing in psychiatry

The field of psychiatric genetics has advanced tremendously over the past 20 years, with high potential for diagnostics, prognosis, and treatment [1, 25, 65]. Several types of genetic approaches have been developed, including copy number variant (CNV) analysis, (targeted) next generation sequencing (NGS), and PRS. Below, we present a brief overview of genetic methodologies with the highest yield and utility within clinical settings in psychiatry.

### Diagnosis and prognosis

With the advent of GWASs, hundreds of new genetic *loci* have been discovered to be associated with various diseases, including psychopathological traits [66] and psychiatric disorders such as anxiety and mood disorders [67, 68], and schizophrenia [69–71]. While genome-wide association analysis itself cannot be used as a test for diagnostic or prognostic purposes at an individual level, it does provide scientific support for individual calculations of PRS.

PRS can be considered as a measure of the cumulative impact of hundreds to thousands of individually weakly associated common genetic variants [72, 73]. As such, PRS is commonly defined as a single value estimate of an individual’s propensity to a phenotype. It is calculated as a sum of their genome-wide genotypes weighted by the corresponding genotype effect sizes from summary statistics GWAS data [72, 73]. While common genetic variants usually only confer a subtle increase in risk for complex phenotypes when examined individually, their cumulative impact expressed in PRS confers a more substantial risk for the disease [8, 74, 75]. Findings from recent studies suggest that PRS may become a useful tool in psychiatry for both diagnostic and prognostic purposes. For example, patients with psychotic symptoms, as well as their relatives, have been found to present significantly higher PRS for schizophrenia and bipolar disorder than healthy controls [34, 76, 77]. PRS has also been shown to be useful in identifying a subset of individuals more likely to relapse and develop schizophrenia among individuals with first-episode psychosis [78–80], patients with schizophrenia likely to be treatment-resistant [81], as well as to be a predictor of antipsychotic effectiveness in individuals with first-episode psychosis [82]. However, several barriers, including low clinical significance, still need to be overcome before PRS can be clinically useful (see section “Barriers to genomic testing in clinical psychiatry settings”) [9, 83].

While the risk for most psychiatric disorders has been shown to be influenced by many common, low-risk variants (as outlined above), rare and highly penetrant variants can also play a role. Even though each rare variant explains only a fraction of disease vulnerability in the population, on an individual level, they confer a much greater risk of developing a certain disorder than the risk predicted by PRS. For example, the risk for ASD in individuals with a 3q29 deletion or a 7q11.23 duplication is estimated to be 38% [84, 85] and 33% [86], respectively. Moreover, when comparing European individuals with ASD to matched controls, cases have been shown to carry a 1.19-fold higher global burden of rare CNVs, rising to a 1.69-fold higher prevalence for *loci* previously implicated in either ASD and/or ID [31]. Finally, the proposed clinical implementations of genetic testing in ASD include the development of new therapeutic strategies and the identification of treatable somatic comorbidities [30, 87, 88].

### Treatment response prediction

Genetic variants, such as single-nucleotide variants (SNVs), have been associated with a higher risk of adverse drug reactions to psychotropic medications, such as antipsychotics and antidepressants [89]. For example, this is the case with clozapine, a second-generation antipsychotic drug indicated for treatment-resistant schizophrenia and useful in other psychotic and mood disorders [90]. Clozapine may induce agranulocytosis, a life-threatening condition that is associated with genetic variation in several genes, including *HLA-DQB1*, *HLA-B*, and *SLCO1B3/SLCO1B7* [91–95]. The subset of patients carrying any of these variants present a risk up to 1175% higher than the overall clozapine-treated population; therefore, performing genetic testing for this variant may be clinically useful in certain situations, e.g., when patients are prescribed clozapine but do not undergo regular blood checks [92, 96–98]. Another scenario where such testing may be of use is in patients diagnosed with 22q11 deletion syndrome. Although this group shows similar clinical improvement after clozapine therapy, seizures and other rare serious side effects are more commonly reported compared to clozapine-treated patients without 22q11 deletion syndrome (OR= 6.5 and OR=22.1, respectively) [99].

Moreover, investigating the clinical usefulness of genetic testing for indications is also relevant for lithium, given the high variability in response, the narrow therapeutic window, the potential severity of side effects, and the associated current underuse of this drug. In the largest lithium response GWAS to date by The International Consortium of Lithium Genetics (ConLiGen), a single locus of four linked SNPs on chromosome 21 was significantly associated with lithium response (all *p* values<5.0×10^−8^) [100]. The same study showed that patients treated with lithium who carried these associated alleles had a significantly lower rate of relapse compared to carriers of the alternate alleles (*p* value=0.03, hazard ratio=3.8) [100]. Another study (using largely the same dataset, based on 14 different sites) evaluated the extent to which lithium response could be predicted based on almost 48,000 genotyped SNPs using machine learning and found that lithium response could be predicted to above-chance levels in two sites of the dataset and in a subset with only those patients that were followed prospectively [101]. However, response could not be predicted in the overall dataset and it was suggested that this was due to heterogeneity arising from multisite data pooling [101].

Furthermore, over 50 cytochrome P450 enzymes are key for the metabolism of several medications, with 90% of all medications being metabolized by six of them, especially *CYP3A4* and *CYP2D6* [102]. *CYP3A4* is implicated in the metabolism of over 50% of commonly prescribed psychotropic drugs, including antipsychotics, antidepressants, anxiolytics, and mood stabilizers [89], and *CYP2D6* enzymes mediate the oxidative metabolism of at least 30 psychotropic medications [103, 104]. Additionally, polymorphisms of their encoding genes have been shown to influence patients’ responses to risperidone and aripiprazole [105, 106], while recent evidence on clozapine hints that not genotype-predicted enzyme activity but rather phenoconversion-predicted enzyme activity (i.e., considering inducers and inhibitors) influences clozapine levels and symptom severity [98].

Finally, clinical guidelines have been developed by the CPIC on the prescription of selective serotonin reuptake inhibitors and tricyclic antidepressants by *CYP2D6* and *CYP2C19* genotypes [37, 38]; atomoxetine by *CYPD26* genotypes [107]; opioid therapy by *CYPD26*, *OPRM1*, and *COMT* genotypes [108]; and carbamazepine and oxcarbazepine by *HLA-A* and *HLA-B* genotypes [109].

## Barriers to genomic testing in clinical psychiatry settings

**Fig. 1:**
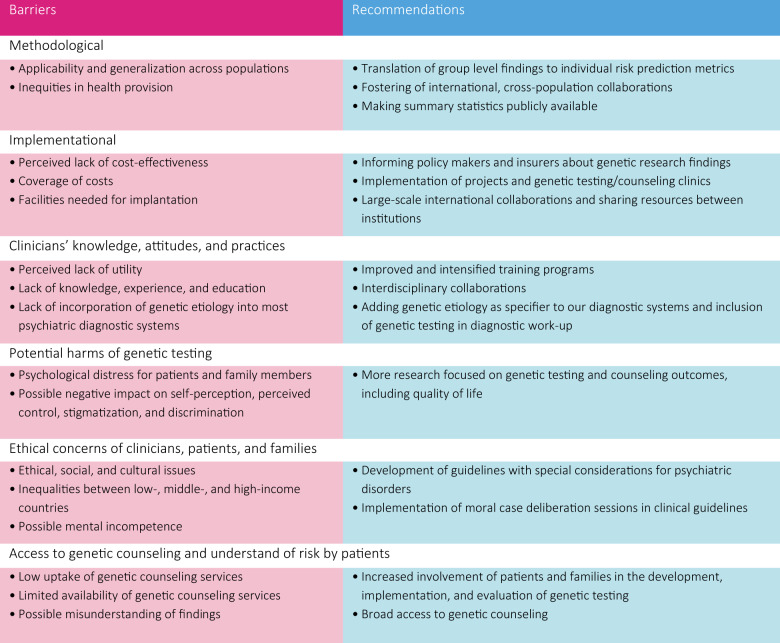
Barriers to genetic testing in clinical psychiatry settings and recommendations on how to overcome them. The first panel lists barriers as grouped in six different categories according to the nature of the barriers (i.e, methodological, implementational, etc.). In the same regard, recommendations are provided for each of the barrier categories.

Although promising, many of the abovementioned techniques and methodologies are not yet ready for direct implementation in the clinic. Below we elaborate on and analyze several barriers to the implementation of genetic testing in clinical psychiatry (Fig. 1), so that they may be more easily overcome, enabling safe and informed genetic testing and potentially setting the stage for precision medicine in psychiatry.

### Methodological

Several methodological challenges currently stand in the way of the applicability of genetic testing at a patient level in psychiatry. First, the effect sizes and the explained variances of PRS at this moment are small, hampering their utility for individual risk prediction [53]. This individual risk prediction is expected to improve by increasing GWAS sample size. However, even (relatively) large effect sizes found to date do not guarantee that PRS will be useful for individual risk prediction. It has recently been shown that PRS for schizophrenia did not improve individual outcome prediction compared with information from a routine psychiatric examination [110]. Thus, to achieve clinical utility, PRS must not only have predictive power, but also provide information that cannot be obtained by conventional means.

Second, there is still uncertainty about whether findings from studies can be extrapolated to people of different ancestries as risk differences attributable to ancestry may differ up to 10-fold [111]. So far, results remain conflicting, e.g., regarding the use of PRS for prognosis prediction in patients with first-episode psychosis. Similar discriminatory power for predicting case-control status and disease course was found in people of European and Brazilian ancestry, while this discriminatory power was considerably lower in people of African ancestry [79, 112]. At the policy level, these issues may raise concerns regarding health inequities as people of non-European ancestry may be at a disadvantage if they cannot also benefit from research, largely derived from European subjects [113, 114]. In addition, some authors even argue that PRS may be a “public health hazard,” criticizing the lack of interpretation of genome-wide association signals at a cellular and physical level [115].

### Implementational

Pharmacoeconomic research has shown conflicting evidence regarding cost-effectiveness of genetic testing [116]. Early studies in major depressive disorder seemed to suggest single gene testing was cost-ineffective [117]; however, more recent, multi-gene, commercially available pharmacogenomic testing has been reported to be cost-effective [118]. Without unequivocal evidence of its cost efficiency, the integration of pharmacogenomic testing in clinical practice will be impeded, as policy makers and other key stakeholders will refuse to provide funding.

In the United States of America (USA), physicians have historically considered funding a considerable barrier to the use of pharmacogenomic testing in clinical practice [119], and for successful implementation, at least genotyping costs must have public or private insurance coverage [5, 120]. Currently, some insurance providers in the USA (such as Managed Medicare and Medicaid) have introduced coverage determinations that enable reimbursement of pharmacogenetic testing, and while the number of claims for coverage of pharmacogenetic testing remains low, it has more than doubled in recent years [121].

Apart from implementation costs, some studies have also identified perceived pragmatic barriers to the implementation of genetic testing, such as infrastructure, human resources, and sustainability [6, 120, 122, 123]. The former would include the required availability of testing facilities that may be accessible to all, as well as the availability of genetic counseling. Genetic testing should be accompanied by the provision of appropriate services ready to explain the implications of testing, perform the testing itself, and provide guidance regarding the test results [124, 125].

### Clinicians’ knowledge, attitudes, and understanding

Studies show that clinicians see the potential benefits of using genetic testing, such as guidance in therapeutic decision-making and a positive impact on patients’ motivation and adherence, but they also mention several barriers [126, 127]. These include a lack of knowledge (not knowing which test to order or not feeling comfortable with interpreting test results), a perceived lack of utility (the results do not alter clinical decision-making), and even potential harmful implications to patients (concerns about the impact on the patients’ employability or insurability) [128]. It would be hard to make a case for genetic testing on an already underserved, stigmatized population such as those with mental illness, when such a procedure would result in a loss of health insurance or employment [129].

Another significant barrier to the adoption of genetic testing is the lack of general understanding of genetics, probability and risk prediction by patients, families, and clinicians themselves [130].

Genetic knowledge is also seen as advancing at an accelerating pace. What is standard practice at the start of a clinician’s residency may already be outdated by the end of it. This rapid change and advancement may cause clinicians, including psychiatrists, to feel uncomfortable making decisions about which tests to order, interpreting the results, and most importantly, communicating such results to patients and families [131].

Finally, genetic etiology has not been incorporated into most psychiatric diagnostic systems, e.g., the Diagnostic and Statistical Manual of Mental Disorders (DSM-5). Classification of most psychiatric disorders, such as schizophrenia, still relies solely on clinical signs and symptoms. Of note, the identification of a ‘medical’ cause is explicitly formulated as an exclusion criterion for most diagnoses, such as schizophrenia. This implies that people who meet the schizophrenia inclusion criteria and have an identified genetic etiology (e.g., 22q11 deletion syndrome) formally cannot be diagnosed with schizophrenia [88].

### Psychological consequences and potential harms

Obtaining genetic risk information may also carry negative consequences for patients and their family members. First, there is the risk that patients and relatives may misinterpret complex genetic information. For example, when it is stated that “addiction is 50% genetic in origin”, this can be understood in several ways. Families may understand that relatives have a 1 in 2 chance of developing a similar disorder or that a lack of positive family history somehow confers immunity [132]. Clearly, both conclusions are false; but the impact of such (common) misconceptions can be dramatic. As the positive perception of genetic testing increases with better understanding, it is essential to provide a clear explanation and confirm that the information has been correctly understood.

Psychological side effects of genetic testing include anticipatory fear and anxiety, particularly when a positive test result is expected and its implications are feared [133, 134]. After receiving a positive genetic test result, patients have been shown to feel as a burden on their families and experience feelings of blame and guilt. This psychological distress affects not only the patient but also family members, who themselves are confronted with a possible increased genetic risk of disease [134]. Self-perception can change negatively after realizing that one is at increased risk for a certain disease, something one may have been previously unconcerned about. Furthermore, given the common perception that genetic risks are immutable, perceived control over the disease, and motivation to change health-related behavior can decrease, secondary to a diminished belief that changing behavior will reduce risks [135, 136].

Lastly, commonly reported concerns with genetic testing include stigmatization and discrimination. Patients with psychiatric disorders are already among the most stigmatized groups in society, which can impair help-seeking and quality of treatment, and can lead to feelings of exclusion [137, 138]. Fear that genetic information will be used for discriminatory purposes by employers and insurance companies also constitutes an important barrier [129].

### Access to genetic counseling

Adequate care after genetic testing, including support groups or psychological follow-up, is pivotal for both patients and relatives to cope with results [139, 140]. This can be achieved by embedding genetic testing in genetic counseling. However, at this point, genetic counselors receive relatively few referrals from psychiatrists, despite the reportedly high demand for psychiatric genetic counseling among people with mental illness [141]. Genetic counselors often do not provide this service to patients with mental illness and while most believe psychiatric genetic counseling may be valuable for both patients and family members, they also doubt the utility [141]. This is mainly due to the perception of genetic counselors that they do not have sufficient psychiatric genetic data, resources and time [141]. These issues are even more pressing in low- and middle-income countries (LMIC), where medical genetics training is even less implemented. Moreover, social and cultural determinants also play a key role in the uptake and understanding of genetic services. It has been argued that religious principles and cultural beliefs can pose barriers to the acceptability and use of genetic services [134]. However, we believe the opposite may also hold: religious traditions and thinking may provide valuable insights when discussing ethical aspects of genetic testing, e.g., regarding coping strategies when dealing with the setback of receiving a genetic diagnosis.

## Recommendations to overcome barriers to genomic testing

Below we outline recommendations to overcome the barriers discussed in the previous section. This is not meant as an extensive list and as new insights develop, undoubtedly new avenues to address such challenges will ensue.

### Education

From medical school to medical specialty training, the acquisition of appropriate genetics knowledge, skills, and attitudes should be encouraged. This is of paramount importance given the role of psychiatrists in providing support and management to patients and families with, or at risk of, highly heritable psychiatric conditions [142]. Such education helps prepare for future clinical advances and should include empowering clinicians to identify patients who could benefit from genetic testing and counseling, to correctly interpret and apply results in clinical practice, and finally, to communicate genetic information in an understandable and nondirective manner [143].

Psychiatrists should always be aware of and assess the emotional, ethical, legal, and social impact of genetic information on patients and their families [128]. This can be further facilitated by interdisciplinary collaboration between general practitioners, medical geneticists, genetic counselors, and psychiatrists, which in turn may increase clinicians’ knowledge and adherence to genetic testing recommendations and improve patient satisfaction [144, 145].

Furthermore, the International Society of Psychiatric Genetics formed a Residency Education Committee to identify key genetic knowledge to be taught in psychiatry training programs [142, 143]. Following this educational guideline may help empower future generations of psychiatrics and ensure adequate implementation of psychiatric genetic testing in clinical settings [4, 146].

On a similar note, training residents in the genetic aspects of mental health would encompass a wide range of clinical benefits. For example, specific training may raise residents’ awareness of genetic risk, allow for community support to patients and families, and facilitate reproductive counseling and family planning to parents with affected children. In addition, training programs may enable residents to make better informed medication choices to reduce the risk of severe medication side effects [142–144].

### Implementation of genetic counseling

Initiatives such as PDGENE [147], an ongoing project aimed at offering both genetic testing and genetic counseling at no cost for people with Parkinson’s disease in North America, are considered potentially useful in increasing not only patients’ access to genetic counseling, but also clinicians’ knowledge about the clinical relevance of test results [148]. Similar initiatives can be implemented in the field of psychiatry, to give patients and clinicians better access to genetic counseling, both on-site and remotely. In 2012, the first specialist psychiatric genetic counseling clinic opened in Canada, which was successful in fulfilling unmet needs of patients and family members with questions about the etiology and recurrence risks of disease and has been shown to enhance empowerment and self-efficacy [149].

It is important to make psychiatric genetic counseling services culturally appropriate, socially and financially accessible, and ethically coherent in order not to further alienate already underserved populations [150]. Especially for LMIC, resources for implementing genetic testing and counseling are currently limited. This could be enhanced by large-scale international collaboration [65, 151–153] and sharing resources between institutions, for example, through university-based exchange programs or government-level collaborations. An example of the latter is Genetic Testing in Emerging Economies (GenTEE), a European Union initiative aimed to inform policy decisions in LMIC on the challenges of delivering equitable access to genetic testing services [154].

### Dissemination

We believe there is also a pressing need to help shape public mental health policies and clinical guidelines, by informing both public health systems and private insurance companies about tests that have shown beneficial clinical applicability, such as pharmacogenomic testing in cases of repeated nonresponse or high susceptibility to side effects. Factors considered by insurers when formulating medical coverage policies for pharmacogenomic testing include availability of clinical guidelines, use by physicians in current clinical practice, cost-effectiveness, and patient interest [5]. Moreover, the most determining factor in coverage is conclusive evidence of positive pharmacogenomic testing for health outcomes [146, 155, 156]. Whenever these conditions are met, insurers and public health systems should consider funding genetic testing. In the past few years, inroads have been made in the US, where pharmacogenetic testing, now covered by several insurance providers, has seen an increasing trend in its uptake [121]. In the Netherlands, the Dutch Pharmacogenetics Working Group [157] has already integrated pharmacogenetic testing into the prescription systems.

### Overcoming implementation barriers

Commercially available pharmacogenetic tests are becoming increasingly accessible due to reduced pricing and simplified implementation procedures [158]. For example, a proposed “evidence-based” genetic testing panel includes a minimum gene and allele set for pharmacogenetic testing in psychiatry that includes 16 variant alleles within five genes (i.e., *CYP2C9*, *CYP2C19*, *CYP2D6*, *HLA-A, HLA-B*) [159]. Such a panel would allow the standardization of protocols to serve as an accompanying tool for clinicians in selecting psychotropic medications and dosing, including antidepressants and mood stabilizers [40, 160, 161].

In addition, some commercially available pharmacogenetic test panels may be well equipped to facilitate the implementation of most pharmacogenomic dosing guidelines relevant to psychiatry, including those associated with *CYP2D6* and *CYP2C19* [159, 161, 162]. However, one should be aware that currently commercially available gene panels show dramatic variability [163]. A standardized, transparent, and systematic evaluation of available evidence is needed to establish this evidence and reduce heterogeneity [159, 163, 164].

Regarding the current lack of integration of genetic etiology in the DSM-5, one way to close this gap is by adding genetic etiology as a specifier to the diagnosis, in addition to the symptom-based diagnostic criteria, as has been suggested for ASD [88]. By including known specifiers in classification systems whilst omitting exclusion criteria such as “attributable to a known medical condition,” clinicians will be encouraged to assess and document genetic and nongenetic etiologies for improved diagnostics [88].

### Bridging the gap between bench and bedside

We also signal a need to leverage the potential of genetic findings for diverse patient populations. The past years have indeed witnessed an increase in GWASs of mixed populations by the Psychiatric Genomics Consortium, as well as the coming into existence of genetic studies in currently underrepresented populations, as exemplified by the Latin America Genomics Consortium. Further advancing such diversity will facilitate greater PRS accuracy in populations of non-European ancestry [112, 113]. By addressing these research (and consequently health) inequities, the full and equitable potential of PRS will also be realized in individuals already underserved by health services [124, 125, 134].

Additionally, it is necessary to translate group level findings to individual risk prediction metrics to increase the clinical relevance of PRS [8, 53, 75, 165]. This can be done by using PPVs as these allow for stratification of individuals into groups with different outcome probabilities and because they depend on both the strength of association and the baseline prevalence [85]. Furthermore, before stratifying the entire population into risk groups, a more feasible goal may be to identify a subset of individuals already at risk for a certain disease, based on genetic factors in combination with clinical risk factors [53]. This may allow for better risk prediction at an individual level, as modest effect sizes conferred by PRS will lead to more substantial differences in absolute risk when applied in populations with a higher prevalence of certain phenotypes (as opposed to the low population prevalence of these phenotypes) [85]. Finally, more research should tackle the lack of current knowledge on the impact on quality of life in patients and their families after genetic testing in the context of psychiatry [140].

### Developing new guidelines

First, we propose an update on current diagnostic guidelines that build on previous efforts, analogous to those published for ASD and ID [166, 167]. A statement on genetic testing is also available from the International Society of Psychiatric Genetics website (last updated in 2019) [168]. Furthermore, treatment guidelines should incorporate pharmacogenomic recommendations from the CPIC clinical guidelines [169] that are already available and further guidelines should be developed as new evidence arises for other drug classes, e.g., antipsychotics. The Dutch Pharmacogenetics Working Group [157] has called for a Europe-wide implementation of its pharmacogenetic guidelines, which would aid in their homologation and widespread use [170].

Moreover, genetic testing and counseling may be included in guidelines of psychiatric associations across the globe [171]. These guidelines should encompass special considerations for situations involving people with psychiatric disorders, including those with impaired mental competence. For example, in such guidelines ethical case deliberation sessions may be suggested for situations where obtaining informed consent is not possible [172]. Procedures should be standardized and should aim to uphold human rights and bioethical principles, while at the same time accounting for cultural differences across the world.

### Empowering patients and families

For successful implementation of clinical genetic testing, it is essential that patients, families, and caretakers’ associations are involved in the process of development, implementation, and evaluation of genetic testing. These key stakeholders should be actively empowered and encouraged to provide voices and input that shape public mental health policy, clinical guidelines, and research proposals. By doing so, barriers to access genomic testing and genetic counseling may be overcome. Genetic counseling for psychiatric disorders has proven to be effective in increasing empowerment in both patients and family members [140, 149, 173]. We recommend that the next step is to make genetic counseling widely available for patients and families. The Genetic Counselling Outcome Scale or its abbreviated version, the Genomics Outcome Scale, may be used to measure patient-reported outcomes when evaluating genetic counseling and testing services [174].

## Conclusions

With the advancement of new genetic testing methodologies, more discoveries can be made at a rapid pace in the field of psychiatric genetics. Several challenges currently hamper the implementation of psychiatric testing, be it broad or more targeted genetic testing in clinical settings. We are optimistic about the implementation of genetic testing in clinical psychiatry around the world as a variety of recommendations can be followed to overcome such barriers. To achieve this, it will be essential that all relevant stakeholders, and especially patients and family, are actively involved. We encourage future research projects to investigate the potential beneficial effects of these recommendations on genetic counseling settings and the quality of life of patients and their relatives around the world.
